# Injection Molded Novel Biocomposites from Polypropylene and Sustainable Biocarbon

**DOI:** 10.3390/molecules24224026

**Published:** 2019-11-07

**Authors:** Mohamed A. Abdelwahab, Arturo Rodriguez-Uribe, Manjusri Misra, Amar K. Mohanty

**Affiliations:** 1 Bioproducts Discovery and Development Centre, Department of Plant Agriculture, Crop Science Building, University of Guelph, 50 Stone Road East, Guelph, ON N1G 2W1, Canada; mabdelwa@uoguelph.ca (M.A.A.); rarturo@uoguelph.ca (A.R.-U.); 2 Department of Chemistry, Tanta University, Tanta 31527, Egypt; 3 School of Engineering, University of Guelph, Thornbrough Building, Guelph, ON N1G 2W1, Canada

**Keywords:** polypropylene, biocomposites, the coefficient of linear thermal expansion, mechanical properties, fillers

## Abstract

Achieving sustainability in composite materials for high-performance applications is a key issue in modern processing technologies. In this work, the structure-property relationships of injection molded polypropylene (PP)/biocarbon composites were investigated with a focus on the thermal properties and specific emphasis on the coefficient of linear thermal expansion (CLTE). Biocomposites were produced using 30 wt.% biocarbon in a PP matrix, and two different sources of biocarbon produced at ~650 and 900 °C were used. The overall results were compared with 30 wt.% glass- and talc-filled PP composites. Due to the lamellar morphology of the talc developed during the extrusion-injection molding processing, talc-filled composites showed an increase in the CLTE in the normal direction (ND), and a reduction in the flow direction (FD) with respect to the neat polymer. Glass fiber composites also showed an improvement in the CLTE with respect to the neat polymer. However, the biocarbon-based composites showed the best properties in the ND, with improved values in biocarbon produced at higher temperature. The FD values for both biocarbon composites were improved with respect to the matrix, while biocarbon created at lower temperature showed slightly lower expansion values. A comprehensive explanation of these overall phenomena is supported by a series of morphological, thermal, mechanical and rheological tests.

## 1. Introduction

The circular economy is a shift towards a more sustainable society. Commodity plastics like polyolefins can still be enhanced in the circular economy [1]. Polypropylene (PP) is a versatile commodity polyolefin widely used in industry for multiple applications. It has low cost, excellent chemical resistance, low density (0.905 g/cm^3^), high UV resistance, and high melt strength and stiffness, as well as tensile strength comparable to low- and high-density polyethylene (PE) [2]. Polypropylene composites, including thermoplastic polyolefins (TPOs), are the most commonly found plastics in the interior parts of vehicles. Short glass fiber and talc are the main fillers/reinforcements used in these systems, but natural fillers (such as natural fibers) are becoming established as promising reinforcements, as they are considered to make an important contribution to weight reduction [3]. Our research group has been working specifically on the characterization of various sources of sustainable biocarbon, and the resulting reports have shown that this material is an efficient additive and/or cost competitive filler for plastics. One fundamental technical advantage of biocarbon with respect to natural fibers is its thermal stability, allowing biocarbon to be processed alongside polymers with high melting points, such as nylon. Biocarbon is produced from the pyrolysis of biomass. The main variables are the temperature of pyrolysis and the residence time in the reactor. Arnold et al. [4] recently reported that the most important variable in this process was the temperature of pyrolysis, rather than the residence time, since higher temperatures allow the production of purer forms of biocarbon and the removal of functional groups (hydroxyl and carboxyl groups). The presence of polar functional groups resulting from lower pyrolysis temperatures contributes to the enhancement of properties when the composites are prepared with more polar polymers, as is the case for nylons [5]. Wang et al. [6] showed that most of the properties of biocarbon-filled PP composites are similar to those of talc-filled composites. Despite the lack of studies related to thermal properties, these authors showed that the particle size and morphology strongly affect the properties of the final product. Furthermore, the results showed similar tensile properties for both talc- and biocarbon-filled composites. The impact strength of biocarbon-based composites was lower when compared to composites manufactured with talc. However, by reducing the particle size of the biocarbon particles, the notched Izod and un-notched impact strength improved and even surpassed the level obtained with mineral filler. In general, the use of biocarbon in composites has shown performance comparable with or superior to neat polymers [7,8].

The CLTE for PP presents different behavior below and above the glass transition temperature (*T*_g_). The effect of fillers on CLTE is more evident above the *T*_g_. The CLTE values are also affected by the properties of the fillers. Kalaitzidou et al. [9] reported similar values for the expansion below the *T*_g_ for carbon black/graphene/carbon fiber composites, and slightly higher when compared to the neat PP. While carbon fiber composites showed higher expansion above the *T*_g_ in the normal direction, graphene composites and carbon black showed lower expansion in both directions (normal direction and flow direction) compared to neat polypropylene and carbon fiber composites. The performance of the graphene particles was attributed to their capacity to form a layered structure during extrusion. While no discussion was offered for the behavior of carbon black composites, it may be associated with the thermal conductivity and the particle size, as well as the shape in carbonaceous materials [9].

Furthermore, Snowdon et al. [10] reported higher CLTE for talc-filled poly(lactic) acid composites than those filled with biocarbon. This may be related to the thermal conductivity of the material, with the final morphology being related to platelet formation after extrusion of talc composites. Weidenfeller et al. [11] found that thermal conductivity increased from 0.27 in PP to 2.5 W/(m K) in 30% talc-filled PP. Surprisingly, the thermal conductivity of 30% copper particles filled PP was 1.25 W/(m K). This was attributed on the one hand to the lack of bridging between copper particles, and on the other to the favorable delamination process of talc particles, which normally occurs during extrusion.

The purpose of this paper was to examine the influence of biocarbon obtained at different pyrolysis temperatures (650 and 900 °C) on the thermal and mechanical characterization of PP composites. Emphasis has been placed on studying the CLTE. Biocarbon was incorporated at 30 wt.% in a PP matrix. Similar experiments were performed with glass fiber and commodity talc; both are normally used in this ratio in PP-filled composites with uses in the automotive manufacturing [12]. Thus, the behavior of the biocarbon composites was compared with commercially PP-filled composites currently used in the automotive manufacturing (glass fiber and talc composites).

## 2. Results and Discussion

### 2.1. Morphological Characterization of PP Composites

**Figure 1:**
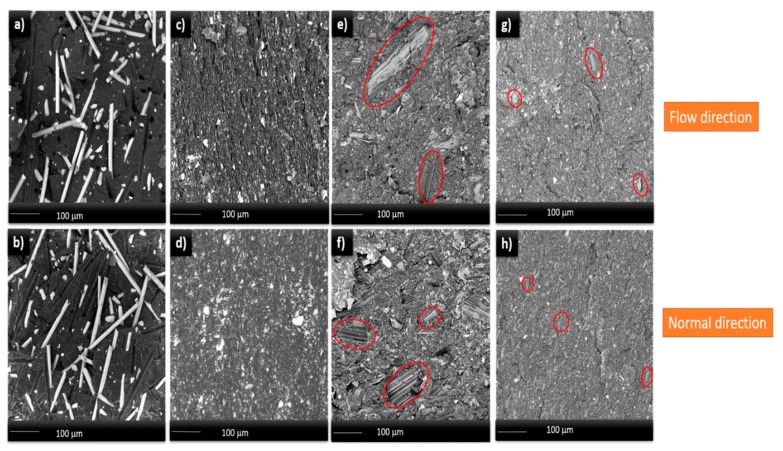
SEM images of PP with 30 wt.% (**a**,**b**) glass fiber; (**c**,**d**) talc; (**e**,**f**) BC 650; and (**g**,**h**) BC900 at 500×. (**a**,**c**,**e**,**f**) in flow direction and (**b**,**d**,**f**,**h**) in normal direction (the red ovals indicate biocarbon particles).

The PP composites were subjected to morphological characterization using SEM and visualization in both normal and flow directions. SEM with low magnification was used to determine the dispersion and orientation of the filler inside the PP matrix. The cryogenically fractured surface of PP with 30 wt.% glass fibers is shown in Figure 1a,b for both normal and flow directions. Both SEM images in the ND and FD show that the glass fibers are aligned in the FD. Some glass fiber pull-outs were observed, although they were not quantified (Figure 1a,b). The same result has been found by other authors [13,14]. Gómez-Monterde et al. [15] observed higher orientation of glass fiber in the FD in similar injection molded samples. Tancrez et al. [16] and Ota et al. [17] observed different orientation in skin and core zones of PP-glass fiber composites resulting from the mold geometry, including its shape and entrance position of the fiber, together with different flow close to the walls of the mold.

The fracture surface of PP with 30 wt.% talc shown in Figure 1c,d is irregular, and the filler particles are mainly parallel to the flow direction. Comparable results have been reported by Wang et al. [6], who blended PP with 27 wt.% talc and observed lamellar or plate-like particles aligned in the flow direction. This phenomenon of talc delamination is associated with better stress distribution during extrusion, yielding high values in the flexural test. Nezhad and Taghizadeh [18] studied the homogeneity and distribution of PP modified by 30 wt.% talc and found that the particles oriented in the FD gave a greater resistance to shrinkage of the matrix in the flow direction than those in the normal direction. Moreover, Branciforti et al. [19] observed that the structure of talc results in the strong *β*-axis orientation of PP crystal in the ND. Figure 1d for ND shows that the platelets of talc particles were homogeneously distributed. 

The fracture surfaces of PP composites with 30 wt.% pyrolyzed biocarbons at two different temperatures, 650 and 900 °C, are shown in Figure 1e–h. The SEM images in Figure 1e,f do not show appreciable difference. A similar observation can be asserted for Figure 1g,h. Furthermore, there is a great difference between the particle size and shape of the two biocarbon samples, which is considered an important factor affecting thermal and mechanical properties, as will be shown by thermal and mechanical characterization. Biocarbon 900 °C particles appear more uniformly dispersed and embedded in the PP matrix. Since biocarbon obtained at higher temperatures shows a reduced presence or absence of functional groups (such as carbonyl and hydroxyl group), this is thought to be the main reason for better dispersion and homogeneity as compared to biocarbon pyrolyzed at 650 °C. Biocarbon particles pyrolyzed at 650 °C have a more elongated shape, which is visible in the figure (red ovals). This is due to the poor adhesion between the partially and/or low-polar pyrolyzation of biocarbon at 650 °C as compared to the non-polar hydrophobic biocarbon pyrolyzed at 900 °C. 

**Figure 2:**
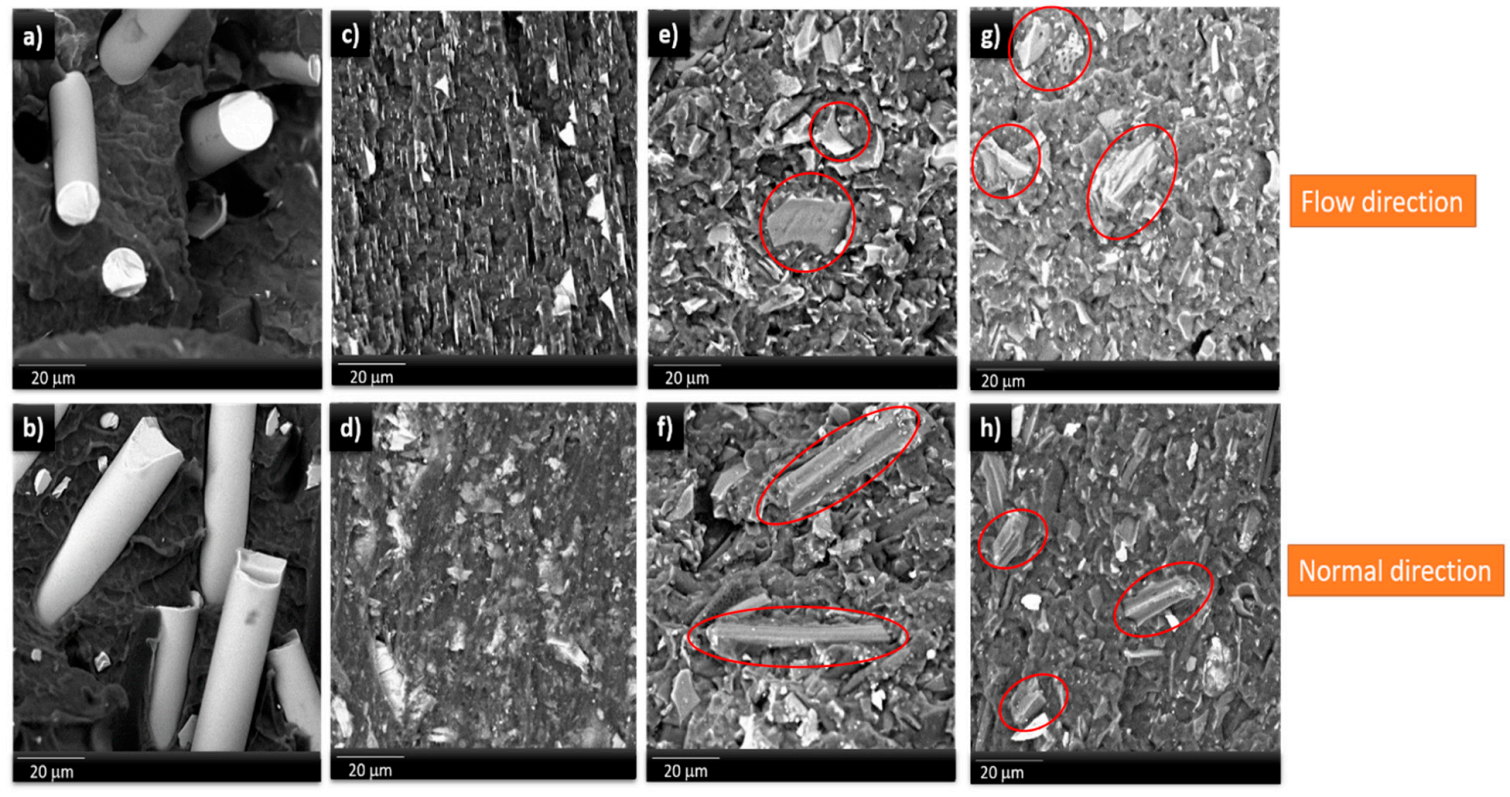
SEM images of PP with 30 wt.% (**a**,**b**) glass fiber; (**c**,**d**) talc; (**e**,**f**) BC 650 and (**g**,**h**) BC900 at 3000×. (**a**,**c**,**e**,**f**) in flow direction and (**b**,**d**,**f**,**h**) in normal direction (the red ovals indicate biocarbon particles).

Figure 2 shows images with higher magnification, taken at the interface of the composites. Figure 2a,b shows images for 30 wt.% glass fiber in ND and FD, respectively. Some glass fibers seem to be separated from the matrix, while some fibers have good contact with the matrix (Figure 2a), which agrees with the findings reported by Wang et al. [20]. 

The 30% talc-filled PP is shown in Figure 2c,d. Talc particles in the composites appear as sticks and plates, showing orientation in the flow direction (Figure 2c). Similar results were observed by Wang et al. [21]. Moreover, Qiu et al. [22] observed that, during injection molding, the orientation of talc platelets is higher in the skin than in the core. These results indicate that the orientation of the particles may affect the thermal properties, in particular the CLTE, as discussed later. On the other hand, biocarbon particles may produce an irregular particle size distribution (Table 1 and Figure S1). Such negative effects can be controlled by using specific methods for particle size reduction such as ball milling. For this reason, the biocarbons used here were ball milled for 1 h. The improvement of the general properties and dispersion within the matrix due to particle size reduction has been confirmed by another research study [23], which showed that the milling of biocarbon resulted in particle size reduction to the nanoscale and increased the surface area. The composites filled with biocarbon are shown in Figure 2e–h. These images show that there is no sign of regular orientation due to the almost elongated shape of biocarbon particles. According to the observation made along ND and FD axes, the length of biocarbon particles seems to be greater in the ND than in the FD due to the difference in the dimensions of the sides of biocarbon particles. In addition, the thickness of the carbon particles in the ND seems to be lower than in the FD. These trends may be reflected in the difference in the CLTE values. Behazin et al. [24] showed that the composites had relatively better interaction (physical interaction) between PP and biocarbon pyrolyzed at high temperatures as compared to biocarbon produced at a lower temperature. Ogunsona et al. [25] showed better adhesion between nylon and biocarbon pyrolyzed at 500 °C than for biocarbon pyrolyzed at 900 °C. This is due to the existence of oxygen-containing functional groups in biocarbon pyrolyzed at 500 °C that can interact with polar nylon polymer. In this study, the increased surface area and absence of polar groups in BC900 enhanced the interfacial adhesion with the PP matrix and led to high reinforcing efficiency, as shown in the mechanical properties, which will be discussed later.

### 2.2. Thermal Characterization of PP-Composites

**Figure 3:**
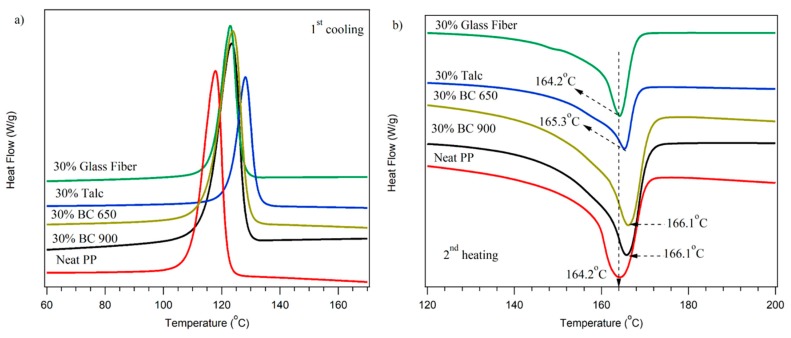
DSC 1st cooling (**a**) and 2nd heating (**b**) of PP and PP-filler composites.

**Figure 4:**
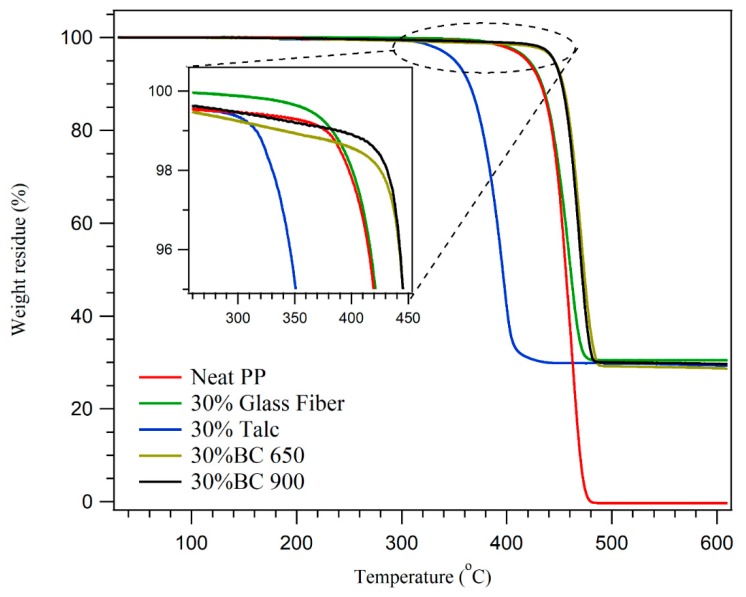
TGA of neat PP and PP-filler composites.

The DSC of the 1st cooling and 2nd heating scans of the composites are shown in Figure 3, and the relevant data are summarized in Table 2. Figure 4 shows the thermal degradation properties of the respective samples. Neat PP and PP composites exhibited only one exothermic peak, as shown in Figure 3. The cold crystallization temperature (*T*_c_) of PP improved with the inclusion of filler, which indicates that the filler acts as a nucleating agent (improving the rate of crystallization). The same effect has been detected by other authors [21,24,26]. The addition of 30 wt.% talc significantly increased the crystallization temperature (increased 10 °C compared to neat PP) due to small spherulites [27]. Neat PP exhibited one melting temperature peak (*T*_m_) at 164.2 °C. The *T*_m_ remained almost constant with the incorporation of 30 wt.% filler. Among the composites studied, PP with 30 wt.% talc or biocarbon had the highest *T*_m_ value of 165.5 °C, as shown in Table 2. This means that talc or biocarbon particles restrict the mobility of PP chains, leading to an increase of the *T*_m_. Thus, the melting temperature in the composite samples was predominantly influenced by the type of filler. The heat of fusion (Δ*H*_m_) was observed to decrease with the inclusion of the filler. Neat PP had a Δ*H*_m_ of 94 J/g, while PP-filler had increase in the Δ*H*_m_ values. This increase in Δ*H*_m_ was due to the addition of filler restricting the movement of the polymer chains and disrupting the regularity of the PP chain structure. Results from Table 2 show that the % crystallinity (*X*_c_) of PP composites increased compared to neat PP, indicating that the growth of PP crystals and the nucleation effect are improved by the incorporation of the filler [27]. The composites containing 30 wt.% filler had approximately 50% crystallinity, which was expected to have an effect in decreasing the CLTE. Snowdon et al. [10] showed that higher crystallinity in polymer composites resulted in higher expansion values in CLTE tests. Thus, the crystallinity is not expected to have a positive influence on the mechanical characterization and CLTE of the composites. 

The influence of biocarbon and different fillers on the thermal stability of the PP was examined using TGA. Figure 4 displays the TGA traces of the composites, and the data is also summarized in Table 2. Neat polymer degraded in one step from 320 to 490 °C with no char residue at 600 °C; however, the PP filler left residual mass (28–30%) relative to their filler contents. *T*_2_ is the temperature at 2% weight loss—a temperature that is critically significant for processing. All PP composites except PP-Talc showed enhancement in their thermal stability compared to neat PP. An interesting result is that the biocarbon-based composites showed better thermal stability than either talc or glass fiber composites. Moreover, the BC900 composites showed enhancement in thermal stability compared to BC650, which indicates the decreasing functionality of biocarbon [28].

### 2.3. Rheological Characterization

**Figure 5:**
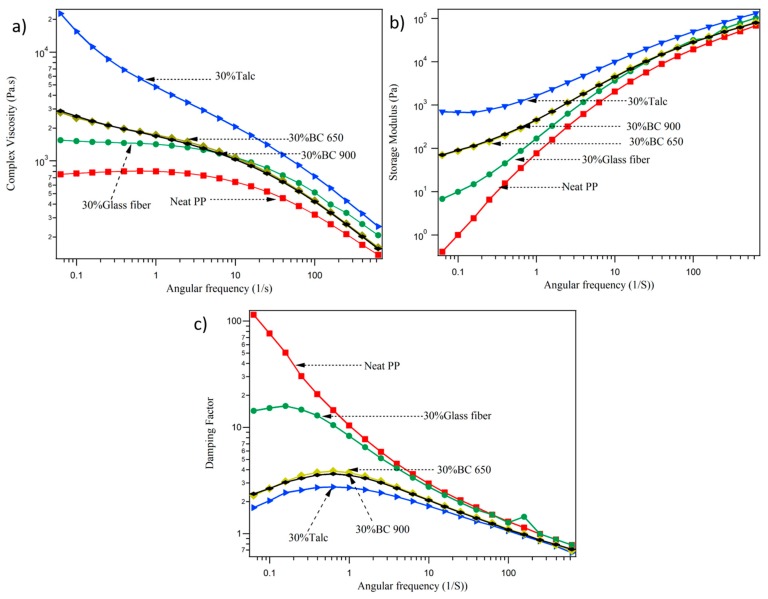
(**a**) Complex viscosity, (**b**) storage modulus, and (**c**) damping factor of PP-filler composites.

The rheological properties of composites are affected via the interactions between the matrix and the filler, size distribution, volume fraction, dispersion, particle size, and the shape of filler. The complex viscosity of neat PP and PP with different fillers is displayed in Figure 5a. Neat PP is a Newtonian flow behavior in which the complex viscosity is constant at lower applied angular frequency. By increasing angular frequency, the complex viscosity of neat PP decreased due to the disentanglement of PP chains, which indicates shear thinning (non-Newtonian flow behavior). As can be observed, the complex viscosity of PP filler composites was higher than for neat PP, which indicates that the filler needs longer relaxation time to flow as well at higher shear stress. PP with 30 wt.% glass fiber showed the same behavior as neat PP with higher complex viscosity. The higher shear stress of PP-Talc during the applied shear force may be due to the orientation of the talc, which disturbs the entanglements of the matrix chains. The complex viscosity of biocarbon composites increased compared to the neat polymer and decreased with increase in angular frequency. There was little difference in complex viscosity between the types of biocarbon used. 

It is obvious that the storage modulus of the composites improved with the addition of filler (Figure 5b). This was due to the existence of rigid filler in the matrix, restricting the movement of PP chains, resulting in an increase in the storage modulus. In the case of PP-30% glass fiber at lower angular frequencies, the PP chains were fully relaxed and revealed typical homopolymer-like terminal performance. At lower angular frequency, the inclusion of talc showed higher storage modulus compared to PP-30 wt.% biocarbon composites. The enhancement in the storage modulus means that talc restricted the polymer chain motion more than biocarbon. There was little difference in the storage modulus between the two types of biocarbon used. 

The damping factor of neat PP and filler composites reflects the effect of prevention or restriction (reduction of segmental motion at the *T*_g_ of polymer chain) from the reinforcing agent on oscillation (Figure 5c). The reduction in the damping factor indicates sufficient adhesion between the polymer and filler. As seen in Figure 5c, the damping factor decreased with the inclusion of the filler, and decreased more with the inclusion of talc and biocarbon. The composites with 30 wt.% talc showed the lowest damping factor and a higher storage modulus when compared to other composites, followed by biocarbon composites, indicating the strong formation of a network structure. This is due to the formation of shear stress between the viscoelastic PP and the filler resulted in the reduction of additional power dissipation in the matrix [29]. The viscoelastic characteristics (complex viscosity, storage modulus and damping factor) of PP filled with different fillers are closely associated with the percolation threshold of the filler network [30]. In other words, the method of preparation of the composites may affect the limits by which a filler can reach percolation or achieve a specific morphology; for example, compression molding vs. injection molding. This effect will be elaborated further in the CLTE and mechanical properties sections.

### 2.4. CLTE of PP Composites

**Figure 6:**
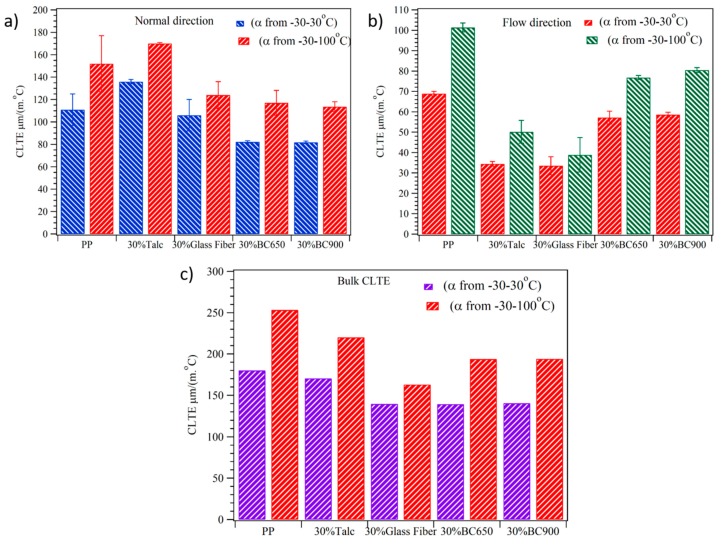
CLTE of PP-filler composites in two different temperature ranges: (**a**) normal direction, (**b**) flow direction, and (**c**) bulk CLTE.

The CLTE of PP-filler composites was calculated in both normal direction (ND) and flow direction (FD) at two different temperature ranges from −30 to 30 °C and from −30 to 100 °C according to ASTM E831-14. Normal direction is perpendicular to the flow of the polymer. Using the same ramp rate, the samples were heated from −60 °C to 150 °C. As observed previously by Lee et al., annealing is an important step for eliminating the irregularities in the CLTE curve normally observed above 60 °C during the 1st scan [31]. Due to the anisotropic effect and helical structure of PP, neat PP shows a non-linear curve (Figure S2), especially at a temperature range of −30 to 100 °C, which led to a higher standard deviation. Moreover, the reduction in the molecular weight during processing that decreases the polymer chain length and the entanglement of the polymer chains may produce variations in the CLTE values. In addition, the intermolecular hydrogen bonds formed by oxygen atoms added to the polymer matrix by thermal oxidation during processing resulted in reduction of the CLTE values [32]. The same observation on CLTE was also made by other authors [31]. As observed in Figure 6, the CLTE values for the ND are higher than that in the FD for both temperature ranges and all samples. Lee et al. observed that the CLTE of PP with different ratios of nanoclay in the ND was slightly higher than that in the FD due to crystallite orientation during processing and polymer matrix orientation being better in the FD than in the ND [31]. 

The CLTE at lower temperature of neat PP in ND was 111 μm/(m. °C) which is comparable to the 93 μm/(m. °C) seen with neat PP in the ND in a slightly different range of 0–30 °C by Kim et al. [33]. The value at −30 to 100 was ~152 μm/(m. °C). However, at −30–100 °C, the CLTE in FD was 101 μm/(m.·°C), which is significantly decreased from the range of −30 to 30 °C (69 μm/(m. °C)). These values confirm the orientation of polymer chains in the FD. The CLTE values at low temperature (−30 to 30 °C) were lower than the values at a higher temperature (−30 to 100 °C), which indicates that by increasing the temperature, the mobility of polymer chain is higher, resulting in an increase in the CLTE values. 

In general, the addition of inorganic fillers or fibers will decrease the thermal expansion of the polymer matrix due to mechanical constraint of the filler [34]. From Figure 6, it can be observed that the CLTE decreased with the inclusion of glass fibers and biocarbon in both ND and FD directions, which indicates that the filler has low CLTE values as compared to the matrix. From Figure 6a, it was observed that, in the ND for both different temperatures, the CLTE of PP with 30 wt.% talc showed slightly higher CLTE than for neat PP. Yoon et al. [35] studied nylon 6 with nanoclay and found that the CLTE in the ND increased with increasing clay loading. This is due to a “squeezing” effect resulting from the orientation of the platelets in the FD direction, which constrains the polymer chains and forms a squeezing force exerted in the ND, thus increasing the CLTE in the ND. The same result was found by Lee et al. [31] in PP blended with nanoclay. However, the CLTE significantly decreased in the FD for both temperatures compared to neat PP (around 50% reduction at both temperatures). Whaling et. al. [36] found that the CLTE decreased with the addition of talc to poly(3-hydroxybutyrate-co-3-hydroxyvalerate) (PHBV) due to the addition of low CLTE particles which decreased the thermal diffusion in the composites. This can be attributed to the mechanical restraint of the PHBV matrix by the inclusion of rigid particles (talc) which restricted the movement of the polymer chains resulting in decreased CLTE. Idumah et al. found a decrease in the CLTE of PP due to the inclusion of exfoliated graphene nanoplates in the presence of Kenaf fiber [37]. This is due to filler particles inhibiting the thermal expansion of PP by restricting mobility of the polymer chains. In this study, it is believed that the reduction in CLTE may be due to the high aspect ratio of the talc, its distribution, and the orientation of the platelets in the flow direction, as confirmed by the SEM micrographs. Moreover, it was observed here that the slight increase in crystallinity resulted in the reduction of the CLTE. This may be explained by the mobility of the PP matrix in a crystal lattice (mean increasing crystallization), leading to a decreased amount of free volume and inhibiting the expansion of the amorphous phase, resulting in reduced CLTE [38]. The inclusion of 30 wt.% biocarbon pyrolyzed at 650 °C significantly reduced the CLTE in ND and FD directions for both temperatures. Codou et al. [28] showed that the inclusion of biocarbon in nylon/PP matrix decreased the CLTE. The reduction of CLTE values is a result of the irregular particle shape of biocarbon. In low aspect ratios of biocarbon, there is no difference in the orientation between ND and FD directions, which leads to CLTE with the same percentage of reduction (around 23%) for both ND and FD at low temperature (−30 to 30 °C). However, at a temperature range from −30 to 100 °C, CLTE decreased by 26% in the ND and 17% in the FD compared to neat PP. As observed in Figure 6b, the CLTE of PP with glass fiber or talc was lower than biocarbon in the FD. Snowdon et al. [10] found that the CLTE of PLA with biocarbon was higher than for PLA/talc due to higher thermal conductivity associated with carbon filler compared to glass fiber or inorganic talc filler. Based on the standard deviation (Figure 6), it can be observed that there is no significant change in the CLTE values between the two different types of pyrolyzed biocarbon (650 and 900 °C) in both the ND and FD. It is believed that there will be a synergistic effect on the mechanical properties of PP, which will be discussed later. The bulk thermal expansion of PP composites was obtained by summing the CLTE for ND and FD (Figure 6c). The bulk CLTE at both temperature ranges showed a significant decrease with the addition of the filler, and depended on the temperature range and orientation of the filler in the case of using glass fiber. Finally, the biocarbon reinforcing agent gives greater enhancement of CLTE in the temperature range of −30 to 100 °C than in the range from −30 to 30 °C. 

### 2.5. Mechanical Characterization of PP Composites

**Figure 7:**
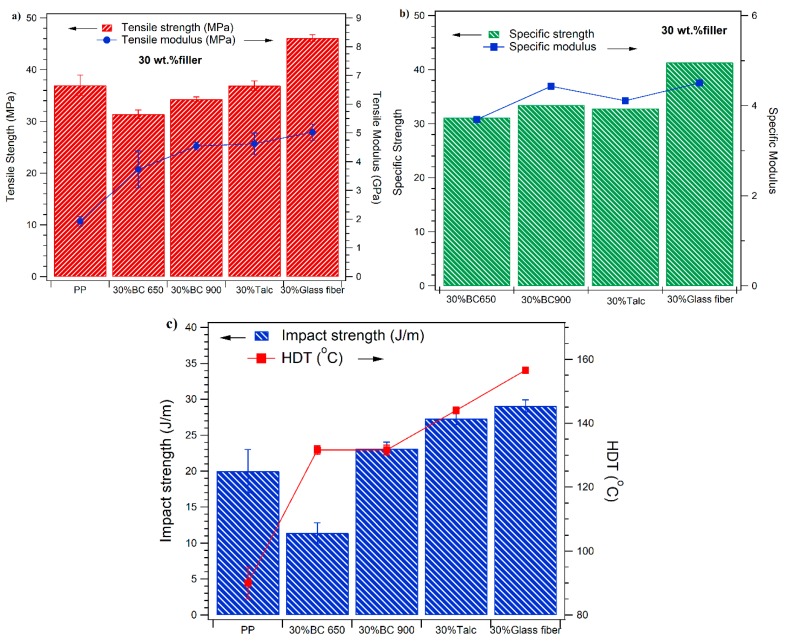
Mechanical and HDT properties of neat PP and PP with 30 wt.% filler: (**a**) tensile and modulus strength, (**b**) specific strength and modulus, and (**c**) impact strength and HDT.

The tensile strength and tensile modulus of the polymer matrix and the composites are presented in Figure 7a. Neat PP presented a tensile modulus and strength of 1.92 GPa and 37 MPa, respectively. Generally, tensile strength is dependent on the stress transfer between the particle filler and the polymer matrix. The addition of 30 wt.% biocarbon (BC) at a pyrolyzing temperature 900 °C or platelet talc produced a minor reduction in tensile strength and enhancement of the tensile modulus. This implies that the filler was able to restrain the PP matrix from deformation. Zhou et al. [39] showed a decrease in the tensile strength of PP/talc composites compared to PP polymer due to the existence of thermal-stresses in the samples during injection molding. Decreasing tensile strength may be due to the difference in CLTE between the matrix and talc or PP matrix and biocarbon, which will lead to induced thermal stresses in the composites. Another possible explanation is that the irregular shape structure of biocarbon (confirmed by SEM in Figure 1 and Figure 2) can produce a compact composite structure by filling the voids due to their small particle size, which can lead to improved stress transfer ability and enhance the tensile strength [40]. However, the addition of pyrolyzed biocarbon at low temperature (650 °C) had lower tensile strength (being decreased by around 15%). A similar trend has recently been reported for the tensile strength of biocarbon in different matrices (polyolefin, polyamide and polycarbonate) [6,24,28,41,42,43,44]. This indicates poor adhesion between the matrix and the low pyrolyzed biocarbon, resulting in decreased tensile strength. However, incorporation of glass fiber showed a positive effect on tensile strength with improvements of around 16% compared to neat polymer. This is owing to the stronger interaction between the filler and the PP polymer, as it is oriented in the FD in the neat polymer (observed by SEM).

The tensile modulus was improved through the incorporation of 30 wt.% filler. For example, the tensile modulus of talc-filled PP composite was improved by 140% with the addition of 30 wt.% talc. The same result was obtained using 30 wt.% pyrolyzed biocarbon at high temperature. A comparable behavior was detected by Behazin et al. [24] for a biocarbon-filled polymer matrix. They found improvements in the tensile modulus of high-temperature pyrolyzed biocarbon (900 °C)-filled PP composites compared to lower-temperature pyrolyzed biocarbon (500 °C) as measured by AFM analysis. This result can be attributed to the higher modulus of biocarbon and the higher turbostratic structure, as well as the presence of increased amounts of minerals in the biocarbon. The improvement of the adhesion between PP and the filler (talc appears as a platelet structure, and biocarbon 900 °C appears as porous particles), as confirmed by SEM, led to better stress transfer to the matrix. The tensile modulus of glass fiber-filled PP composites significantly increased by around 161% for glass fiber composite compared to neat PP. The performance of PP with glass fiber composites was mainly due to the higher stiffness of the fiber and the improvement of the interaction among the matrix and the filler. The same effect has been reported in many different papers [45,46,47,48]. 

The specific flexural strength and modulus were calculated based on the density of the neat PP and composites, and they are displayed in Figure 7b. These properties are very important for materials used in automotive applications. The specific modulus of biocarbon pyrolyzed at high temperature is slightly higher than that of talc-filled PP, and is equivalent to the data obtained from glass fiber-filled PP. The biocarbon 900 °C-filled PP had slightly higher specific strength then talc-filled PP composites, while the biocarbon composites showed lower flexural specific strength compared to glass fiber filled PP. This is a result of better adhesion among the glass fiber and the polymer matrix.

Generally, the inclusion of filler in the polymer matrix reduced the impact strength. The notched Izod impact strengths of PP filled with different fillers are displayed in Figure 7c. The impact strength effect of PP-glass fiber was observed to be higher than that of all other composites and higher than that of neat PP. This was due to the fact that the glass fiber can absorb more energy and hinder crack propagation through impact fracture [47]. Subsequently, this energy loss causes improved impact strength. The impact strength of PP-BC 650 °C significantly decreased compared to neat PP. The larger particle size of the filler which has less surface area led to debonding within the matrix and decreased the impact strength [49]. The variations in the degree of encapsulation of the particles resulted in remarkable contrasts in the impact strength of the PP filled with different fillers. However, the biocarbon pyrolyzed at high-temperature composites showed a higher impact strength than the lower-temperature pyrolyzed biocarbon composites. This may be due to the enhancement of the compatibility between the nonpolar BC 900 °C and the nonpolar PP polymer matrix giving rise to greater resistance to crack propagation.

### 2.6. Dynamic Mechanical Analysis and HDT Measurements

**Figure 8:**
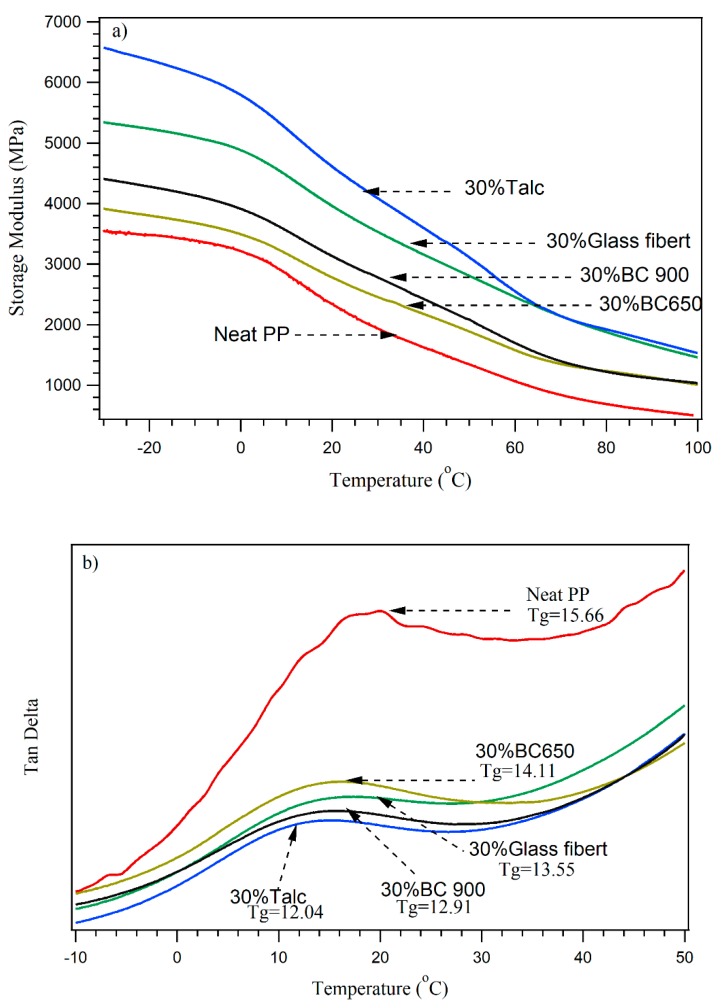
(**a**) Storage modulus and (**b**) tan δ of neat PP and PP-filler composites.

The dynamic mechanical analysis (DMA) results are presented as curves of storage modulus and tan δ (Figure 8a,b). The storage moduli of the matrix and PP-filled composites decreased with increasing temperature. Neat PP had a storage modulus of 2133 MPa at 25 °C, and this increased to 2605 MPa and 2943 MPa with the inclusion of biocarbon 650 and 900 °C, respectively. With the inclusion of glass fiber and talc, it increased to 3733 MPa and 4336 MPa, respectively. Enhancement of the storage modulus of PP-filled with different fillers indicates that the filler restricts the molecular mobility of PP chains at the interface. 

The effects of different fillers on the tan δ shift of the composites are shown in Figure 8b. Neat PP had a tan δ peak at 15.7 °C corresponding to the glass transition temperature of PP. The tan δ of PP-filler composites shifted to a lower temperature with the existence of the filler. For all filler composites, the drop in *T*_g_ was around 1.5–2.5 °C in comparison to neat PP, which is not a significant difference. 

The HDT (heat deflection temperature) is a critical parameter in the automotive industry for product design. The HDT data of PP-filled with different fillers are presented in Figure 7c. The HDT of PP was calculated to be 90 °C. Several factors affect the enhancement of HDT such as the stiffness of the material, the glass transition temperature and the degree of crystallinity [50]. The increase of these parameters due to the addition of filler enhances the HDT of the composites. As observed based on the mechanical properties and DSC, the stiffness and degree of crystallinity increased with the addition of filler (*X_c_* increased by around 3–5% compared to neat PP). Therefore, the HDT data of PP-filler composites followed a similar trend to tensile modulus. PP-glass fiber showed the highest HDT value at around 153 °C, increasing by 63 °C compared to neat PP. However, the inclusion of 30 wt.% biocarbon with two different pyrolyzing temperatures only increased the HDT by around 42 °C compared to neat PP. This result indicates that the filler restricts the chain mobility of the matrix and prevents the polymer chains from deforming under load, which results in enhancing the HDT [44,51].

## 3. Experimental

### 3.1. Materials

Polypropylene homopolymer (PP), with the trade name PP1350N, was purchased from Pinnacle Polymers, USA. According to the technical data sheet, the PP has a high MFI of 55 g/10 min and *d =* 0.9 g/cm^3^. Miscanthus biocarbons were kindly supplied by Competitive Green Technologies (Leamington, ON, Canada). These biocarbons were produced at two different pyrolysis temperatures of 650 °C (BC650) and 900 °C (BC900) for around 20 min under limited oxygen. Since the biocarbon produced tends to retain the shape of the original particles, it was hammer milled and screened by using a 1/64-inch mesh (≤ 400 μm) in order to produce a fine powder. The pyrolysis and hammer mill/screening process, physical properties, morphology, TGA and FTIR data of both pyrolyzed biocarbons have been described elsewhere [6,28,41,52]. Briefly, the pyrolysis was performed in the absence of oxygen in a Metamag unit with built-in conveyer (London ON, Canada), which was the property of Competitive Green Technologies, Leamington, ON, Canada. To homogenize the biocarbon, both samples were subjected to a further particle size homogenization using a ball mill, Pulverisette 5, Pittsboro, NC, USA, with a milling time of 1 h at 200 rpm. For this process, 30 grams of biocarbon, together with 200 1.0 cm diameter ceramic balls, were placed in a 500 mL ceramic jar. The biocarbon (BC) had a density of between 1.3 and 1.4 g/cm^3^, an aspect ratio of ~0.5 and an average particle size measured by SEM of ~3.5 μm (Table 1 and Figure S1). The talc used was high aspect ratio T84 acquired from Imerys Talc America, Inc., San José, CA., USA. Chopped glass fiber was obtained from PPG Fiberglass (HP3273) with an average strand length of 4.5 mm and a filament diameter of 14 μm.

### 3.2. Preparation of PP-Filler Composites

Biocarbon (BC) was dried at 105 °C for 48 h to decrease the moisture content to around 1 wt.% as measured by a moisture analyzer (Sartorious, Atkinson, NH, USA). Neat PP and 30 wt.% filler composites (total weight 10 g) were melt extruded and injection molded using a DSM Xplorer® (micro-compounder, Sittard, The Netherlands, L/D ratio of 150:18 mm,). Extrusion was carried out at 180 °C and 100 rpm screw speed with 2 min residence time and then transferred to a DSM micro instrument for injection molding. Standard samples were produced with a total injection time of 20 secs and 10 bar average pressure. The mold temperature was 30 °C. Finally, the samples were conditioned for 48 h at R.T. before testing. The PP-filler composites containing 30 wt.% filler were designated as shown in Table 3.

## 4. Characterization of PP-Filled Composites

**Scheme 1:**
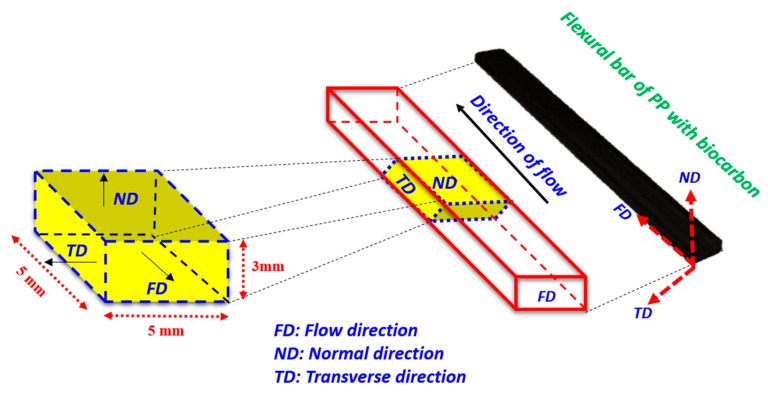
Schematic diagram of the flexural test bar cutting for CLTE measurement.

The morphology of the PP-filled composites was observed by scanning electron microscopy (SEM) with a Phenom ProX (Phenom-World B.V., Eindhoven, The Netherlands) at 15 kV. The specimens were cryo-fractured under liquid nitrogen and mounted on aluminum stubs for imaging. SEM characterization was done on fracture surfaces obtained in two different directions, normal direction (ND) and flow direction (FD) (Scheme 1).

The CLTE of neat PP and PP composites was characterized according to ASTM E831-14 using a thermomechanical analyzer (TMA) (TA Instrument, TMA Q400, New Castle, DE, USA). The specimens with dimensions of width ~5 mm, height ~5 mm and thickness ~3 mm were prepared from the central part of flexural bars. To remove the thermal history, each sample was heated to 80 °C at a rate 5 °C/min and held for 5 min then cooled back down to −40 ℃ and held for 2 min [35]. The specimens were tested in two orthogonal directions, ND and FD, using an expansion probe with a force of 0.05 mN at a heating rate of 5 °C/min (Scheme 1). CLTE was measured over two temperature ranges from −30 to 30 °C and from −30 to 100 °C for both directions after removing the sample thermal history.

The mechanical characteristics (tensile tests) were achieved according to ASTM D638 (Type IV) using an Instron Universal Testing Machine at 5 mm/min for composite samples and 50 mm/min for neat PP. The notched Izod impact properties were achieved according to ASTM D256 by an TMI impact tester (Testing machine Inc. TMI 43-02). The samples were notched immediately after processing. All the mechanical data was achieved from a total of five samples for each material.

Thermal analysis (DSC and TGA) were accomplished with DSC Q 200 and TGA Q500 analyzers (TA Instruments), respectively, at a heating rate of 10 °C/min. All tests were conducted under a nitrogen atmosphere with a flow rate of 40 and 60 mL/min for DSC and TGA, respectively. The 1st heating scan in the DSC was performed to erase any thermal history of the composites. The 2nd heating cycle was selected in order to overcome the crystallinity related to the thermal background of the polymer (processing). The 2nd heating scan was used to evaluate melting temperature (*T*_m_) and crystallinity (*X*_c_). The degree of crystallinity, *X*_c_, was calculated from DSC curves as follows Equation (1):

(1) $$X_{c}=\frac{\Delta Hm\;}{\left(1-ø\right)\Delta Hm^{*}}\times 100$$

where ΔH_m_ is the melting enthalpy (J/g) calculated from the fusion peak of the DSC curve, ΔH_m_* is the theoretical heat of fusion for 100% crystallized PP (207.1 J/g) and ø is the weight fraction of the filler in the composites (30 wt.%) [53]. However, the TGA was used to evaluate the thermal stability at T_2_ (temperature at 2% weight loss) and the char yield (R600) at 600 °C for neat PP polymer and PP-composite samples.

Heat deflection temperature (HDT) and DMA was tested in a Dynamic Mechanical Analysis (DMA Q800, TA Instruments, Inc.) following ASTM D648-18 at stress 0.45 MPa. The neat polymer and PP-filler composites were heated from room temperature until the deflection of 250 μm was obtained at a rate of 2 °C/min and tested using a three-point bending configuration.

DMA was performed via a DMA Q800. The samples were tested at oscillating amplitude of 15 μm and at a frequency of 1 Hz. The neat PP and PP-filler composites samples were heated from −100 to 120 °C at a rate of 3 °C/min using a dual cantilever clamp. The DMA was used to calculate the storage modulus and Tan δ for homopolymer and composite samples.

The complex viscosity and storage modulus of homopolymer and composite samples were performed using an Anton Paar Rheometer (MCR-302). The data were performed at 180 °C with parallel plate geometry (1 mm gap and 25 mm diameter plates) with a strain of 1% (in the linear viscoelastic) and a frequency range between 0.05 and 600 rad/s. All the thermal and DMA data was achieved from a total of two samples for each material.

The densities of neat PP and composite samples were measured via a densimeter (Alfa Mirage, MD-300S, Osaka, Japan). All densities data was achieved from a total of three samples for each material.

## 5. Conclusions

Through this work, PP biocarbon composites showed a significant reduction in CLTE in the normal direction, as compared to the neat PP polymer- and talc-based composites. In the flow direction, talc-based composites showed a better performance as compared to biocarbon-based composites. The bulk CLTE of PP-biocarbon composites exhibited decreased CLTE values compared to talc composite, and equivalent results in the glass fiber composites. SEM photos showed that biocarbons have more elongated particles. However, talc particles can delaminate in a process such as extrusion and, therefore, can form continuous overlapping layers that will expand more in the normal direction in the presence of heat. The *T*_c_ of PP filled with different fillers shifted to a higher temperature compared to neat PP, which indicates that the filler acts as a nucleating agent. Moreover, the *T*_m_ remained almost constant with increasing in the % of crystallinity after incorporation of 30 wt.% filler. The viscoelastic characteristics of PP filled with different fillers are closely associated with the percolation threshold of the filler network. Incorporation of 30 wt.% glass fiber showed the highest mechanical properties compared to any other fillers. Interestingly, composites prepared with biocarbon pyrolyzed at higher temperature (900 °C) showed higher HDT and mechanical characteristics (impact strength, tensile modulus and tensile strength) compared to biocarbon pyrolyzed at lower temperature (650 °C). This can be attributed to the absence of functional groups, which resulted in a greater affinity with the PP matrix. These results show that the overall properties of composites can be modified by using different filler morphologies and that the most important properties of PP biocarbon can be achieved by controlling the temperature of pyrolysis to produce sustainable composites.

## Figures and Tables

**Table 1:** Structural characterization of the biocarbon powder after ball-milling obtained by scanning electron microscopy. The particle size population and aspect ratio were measured by image-based particle size.

Sample Name	Particle Size Population (%)						Aspect Ratio
	< 2 µm	2–3 µm	3–5 µm	5–10 µm	≥ 10 µm	Particle Size Average (µm)	
**BC650**	5.19	24.07	46.0	22.0	2.59	4.26	~0.50
**BC900**	13.05	27.45	40.0	18.13	1.35	3.81	~0.52

**Table 2:** Thermal properties of neat PP and PP-filler composites from TGA and DSC curves.

Code	*T*_2_ (°C)	R_600_ (%)	*T*_c_ (°C)	*T*_m_ (°C)	Δ*H*_m_ (J/g)	*X*_c_ (%)
Neat PP	398 ± 0.34	0.0 ± 00	117.4 ± 0.9	164.2 ± 0.1	94 ± 6	45 ± 3
30% Glass Fiber	400 ± 1.4	29.72 ± 1.0	122.2 ± 1	164.6 ± 0.6	69 ± 3	48 ± 2
30% Talc	340 ± 7.9	29.28 ± 0.2	127.8 ± 0.6	165.5 ± 0.3	73 ± 5	50 ± 4
30% BC650	425 ± 0.18	28.62 ± 0.12	123.8 ± 0.2	165.5 ± 0.9	72 ± 0.7	50 ± 0.5
30% BC900	431 ± 0.3	29.3 ± 0.47	123.5 ± 0.1	165.5 ± 0.5	72 ± 2	50 ± 0.5

*T*_2_ is the temperature at 2% weight loss; R_600_ is the residual weight percentage at 600 °C; *T*_m_ is the melting temperature, Δ*H*_m_ is the enthalpy of melting, and *X*_c_ is the degree of crystallinity.

**Table 3:** Formulations prepared to contain 30 wt.% filler.

Code	PP	Filler
PP	100	/
30%BC650	70	30% biocarbon pyrolyzed at 650 °C then ball milled 1h at 200 rpm
30%BC900	70	30% biocarbon pyrolyzed at 900 °C then ball milled 1h at 200 rpm
30%Talc	70	30 wt.% Talc
30%Glass fiber	70	30 wt.% Glass fibers

## Supplementary Materials

The following are available online, Figure S1: Particle size distribution of biocarbon ball-milled at two different pyrolysis temperature (650 and 900 °C) obtained by image-based particle size analysis, Figure S2: Typical thermal expansion behavior of neat PP in the ND during the second heating runs.

## References

[1] Stahel W.R. (2016). The circular economy. Nature.

[2] Patil A., Patel A., Purohit R. (2017). An overview of Polymeric Materials for Automotive Applications. Mater. Today Proc..

[3] Joshi S.V., Drzal L.T., Mohanty A.K., Arora S. (2004). Are natural fiber composites environmentally superior to glass fiber reinforced composites?. Compos. Part A Appl. Sci. Manuf..

[4] Arnold S., Rodriguez-Uribe A., Misra M., Mohanty A.K. (2018). Slow pyrolysis of bio-oil and studies on chemical and physical properties of the resulting new bio-carbon. J. Clean. Prod..

[5] Usuki A., Koiwai A., Kojima Y., Kawasumi M., Okada A., Kurauchi T., Kamigaito O. (1995). Interaction of nylon 6-clay surface and mechanical properties of nylon 6-clay hybrid. J. Appl. Polym. Sci..

[6] Wang T., Rodriguez-Uribe A., Misra M., Mohanty A.K. (2018). Sustainable Carbonaceous Biofiller from Miscanthus: Size Reduction, Characterization, and Potential Bio-composites Applications. BioResources.

[7] Das O., Kim N.K., Sarmah A.K., Bhattacharyya D. (2017). Development of waste based biochar/wool hybrid biocomposites: Flammability characteristics and mechanical properties. J. Clean. Prod..

[8] Das O., Sarmah A.K., Bhattacharyya D. (2016). Biocomposites from waste derived biochars: Mechanical, thermal, chemical, and morphological properties. Waste Manag..

[9] Kalaitzidou K., Fukushima H., Drzal L.T. (2007). Multifunctional polypropylene composites produced by incorporation of exfoliated graphite nanoplatelets. Carbon.

[10] Snowdon M.R., Mohanty A.K., Misra M. (2017). Examination of a Biobased Carbon Nucleating Agent on Poly(lactic acid) Crystallization. J. Renew. Mater..

[11] Weidenfeller B., Höfer M., Schilling F.R. (2004). Thermal conductivity, thermal diffusivity, and specific heat capacity of particle filled polypropylene. Compos. Part A Appl. Sci. Manuf..

[12] Mohanty A.K., Misra M., Rodriguez-Uribe A., Vivekanandhan S. (2017). Hybrid Sustainable Composites and Methods of Making and Using Thereof.

[13] Kim E.G., Park J.K., Jo S.H. (2001). A study on fiber orientation during the injection molding of fiber-reinforced polymeric composites: (Comparison between image processing results and numerical simulation). J. Mater. Process. Technol..

[14] Kamal M.R., Song L., Singh P. (1986). Measurement of fiber and matrix orientations in fiber reinforced composites. Polym. Compos..

[15] Gómez-Monterde J., Sánchez-Soto M., Maspoch M.L. (2018). Microcellular PP/GF composites: Morphological, mechanical and fracture characterization. Compos. Part A Appl. Sci. Manuf..

[16] Tancrez J.-P., Pabiot J., Rietsch F. (1996). Damage and fracture mechanisms in thermoplastic-matrix composites in relation to processing and structural parameters. Compos. Sci. Technol..

[17] Ota W.N., Amico S.C., Satyanarayana K.G. (2005). Studies on the combined effect of injection temperature and fiber content on the properties of polypropylene-glass fiber composites. Compos. Sci. Technol..

[18] Shelesh-Nezhad K., Taghizadeh A. (2007). Shrinkage behavior and mechanical performances of injection molded polypropylene/talc composites. Polym. Eng. Sci..

[19] Branciforti M.C., Oliveira C.A., de Sousa J.A. (2010). Molecular orientation, crystallinity, and flexural modulus correlations in injection molded polypropylene/talc composites. Polym. Adv. Technol..

[20] Wang K., Guo M., Zhao D., Zhang Q., Du R., Fu Q., Dong X., Han C.C. (2006). Facilitating transcrystallization of polypropylene/glass fiber composites by imposed shear during injection molding. Polymer.

[21] Wang K., Bahlouli N., Addiego F., Ahzi S., Rémond Y., Ruch D., Muller R. (2013). Effect of talc content on the degradation of re-extruded polypropylene/talc composites. Polym. Degrad. Stab..

[22] Qiu F., Wang M., Hao Y., Guo S. (2014). The effect of talc orientation and transcrystallization on mechanical properties and thermal stability of the polypropylene/talc composites. Compos. Part A Appl. Sci. Manuf..

[23] Peterson S.C., Jackson M.A., Kim S., Palmquist D.E. (2012). Increasing biochar surface area: Optimization of ball milling parameters. Powder Technol..

[24] Behazin E., Misra M., Mohanty A.K. (2017). Sustainable biocarbon from pyrolyzed perennial grasses and their effects on impact modified polypropylene biocomposites. Compos. Part B Eng..

[25] Ogunsona E.O., Misra M., Mohanty A.K. (2017). Impact of interfacial adhesion on the microstructure and property variations of biocarbons reinforced nylon 6 biocomposites. Compos. Part A Appl. Sci. Manuf..

[26] Das O., Bhattacharyya D., Hui D., Lau K.-T. (2016). Mechanical and flammability characterisations of biochar/polypropylene biocomposites. Compos. Part B Eng..

[27] Ferrage E., Martin F., Boudet A., Petit S., Fourty G., Jouffret F., Micoud P., De Parseval P., Salvi S., Bourgerette C. (2002). Talc as nucleating agent of polypropylene: Morphology induced by lamellar particles addition and interface mineral-matrix modelization. J. Mater. Sci..

[28] Codou A., Misra M., Mohanty A.K. (2018). Sustainable biocarbon reinforced nylon 6/polypropylene compatibilized blends: Effect of particle size and morphology on performance of the biocomposites. Compos. Part A Appl. Sci. Manuf..

[29] Essabir H., Elkhaoulani A., Benmoussa K., Bouhfid R., Arrakhiz F.Z., Qaiss A. (2013). Dynamic mechanical thermal behavior analysis of doum fibers reinforced polypropylene composites. Mater. Des..

[30] Lisunova M.O., Mamunya Y.P., Lebovka N.I., Melezhyk A.V. (2007). Percolation behaviour of ultrahigh molecular weight polyethylene/multi-walled carbon nanotubes composites. Eur. Polym. J..

[31] Lee H.-S., Fasulo P.D., Rodgers W.R., Paul D.R. (2006). TPO based nanocomposites. Part 2. Thermal expansion behavior. Polymer.

[32] Yang L., Thomason J.L., Zhu W. (2011). The influence of thermo-oxidative degradation on the measured interface strength of glass fibre-polypropylene. Compos. Part A Appl. Sci. Manuf..

[33] Kim D.H., Fasulo P.D., Rodgers W.R., Paul D.R. (2007). Structure and properties of polypropylene-based nanocomposites: Effect of PP-g-MA to organoclay ratio. Polymer.

[34] Holliday L., Robinson J. (1973). Review: The thermal expansion of composites based on polymers. J. Mater. Sci..

[35] Yoon P.J., Fornes T.D., Paul D.R. (2002). Thermal expansion behavior of nylon 6 nanocomposites. Polymer.

[36] Whaling A., Bhardwaj R., Mohanty A.K. (2006). Novel Talc-Filled Biodegradable Bacterial Polyester Composites. Ind. Eng. Chem. Res..

[37] Idumah C.I., Hassan A. (2016). Characterization and preparation of conductive exfoliated graphene nanoplatelets kenaf fibre hybrid polypropylene composites. Synth. Met..

[38] Grestenberger G., Potter G.D., Grein C. (2014). Polypropylene/ethylene-propylene rubber (PP/EPR) blends for the automotive industry: Basic correlations between EPR-design and shrinkage. Express Polym. Lett..

[39] Zhou Y., Rangari V., Mahfuz H., Jeelani S., Mallick P.K. (2005). Experimental study on thermal and mechanical behavior of polypropylene, talc/polypropylene and polypropylene/clay nanocomposites. Mater. Sci. Eng. A.

[40] Guoqiang L., Jack E.H., Su-Seng P., Kurt S. (2001). Analytical modeling of tensile strength of particulate-filled composites. Polym. Compos..

[41] Andrzejewski J., Misra M., Mohanty A.K. (2018). Polycarbonate biocomposites reinforced with a hybrid filler system of recycled carbon fiber and biocarbon: Preparation and thermomechanical characterization. J. Appl. Polym. Sci..

[42] Behazin E., Misra M., Mohanty A.K. (2017). Sustainable Biocomposites from Pyrolyzed Grass and Toughened Polypropylene: Structure–Property Relationships. ACS Omega.

[43] Ogunsona E.O., Misra M., Mohanty A.K. (2017). Influence of epoxidized natural rubber on the phase structure and toughening behavior of biocarbon reinforced nylon 6 biocomposites. RSC Adv..

[44] Ogunsona E.O., Codou A., Misra M., Mohanty A.K. (2018). Thermally Stable Pyrolytic Biocarbon as an Effective and Sustainable Reinforcing Filler for Polyamide Bio-composites Fabrication. J. Polym. Environ..

[45] Yichuan C., Xinyu W., Min N., Qi W. (2017). Preparation of polypropylene/glass fiber composite with high performance through interfacial crystallization. J. Vinyl Addit. Technol..

[46] Xiaofei Y., Hua S., Lichao Y., Hiroyuki H. (2017). Polypropylene–glass fiber/basalt fiber hybrid composites fabricated by direct fiber feeding injection molding process. J. Appl. Polym. Sci..

[47] Rahmanian S., Thean K.S., Suraya A.R., Shazed M.A., Mohd Salleh M.A., Yusoff H.M. (2013). Carbon and glass hierarchical fibers: Influence of carbon nanotubes on tensile, flexural and impact properties of short fiber reinforced composites. Mater. Des..

[48] Ashori A., Menbari S., Bahrami R. (2016). Mechanical and thermo-mechanical properties of short carbon fiber reinforced polypropylene composites using exfoliated graphene nanoplatelets coating. J. Ind. Eng. Chem..

[49] Sudár A., Renner K., Móczó J., Lummerstorfer T., Burgstaller C., Jerabek M., Gahleitner M., Doshev P., Pukánszky B. (2016). Fracture resistance of hybrid PP/elastomer/wood composites. Compos. Struct..

[50] McKeen L.W. (2008). 7—Polyolefins and Acrylics. Effect of Temperature and other Factors on Plastics and Elastomers.

[51] Ogunsona E.O., Manjusri M., Mohanty A.K. (2017). Sustainable biocomposites from biobased polyamide 6,10 and biocarbon from pyrolyzed miscanthus fibers. J. Appl. Polym. Sci..

[52] Behazin E., Ogunsona E., Rodriguez-Uribe A., Mohanty A.K., Misra M., Anyia A.O. (2016). Mechanical, Chemical, and Physical Properties of Wood and Perennial Grass Biochars for Possible Composite Application. Bioresources.

[53] Velasco J.I., Morhain C., Martínez A.B., Rodríguez-Pérez M.A., de Saja J.A. (2002). The effect of filler type, morphology and coating on the anisotropy and microstructure heterogeneity of injection-moulded discs of polypropylene filled with aluminium and magnesium hydroxides. Part 2. Thermal and dynamic mechanical properties. Polymer.

