# Supplementary material for: Injection Molded Novel Biocomposites from Polypropylene and Sustainable Biocarbon
Source: Molecules. 2019 Nov 7;24(22):4026. doi: 10.3390/molecules24224026 (PMC6891267; doi:10.3390/molecules24224026)
Supplement: Supplementary file 1 [file molecules-24-04026-s001.pdf]

Injection molded novel biocomposites from polypropylene and sustainable biocarbon

Mohamed A. Abdelwahab[1,2], Arturo Rodriguez-Uribe[1], Manjusri Misra[1, 3], Amar K. Mohanty[1, 3*]*

1. Bioproducts Discovery and Development Centre, Department of Plant Agriculture, Crop Science Building, University of Guelph, 50 Stone Road East, Guelph, N1G 2W1 Ontario, Canada

2. Department of Chemistry, Tanta University, Tanta, 31527, Egypt

3. School of Engineering, University of Guelph, Thornbrough Building, Guelph, N1G 2W1, Ontario, Canada

*Correspondence to M. Misra, Email: mmisra@uoguelph.ca and Amar K. Mohanty. Email: mohanty@uoguelph.ca

Supporting information

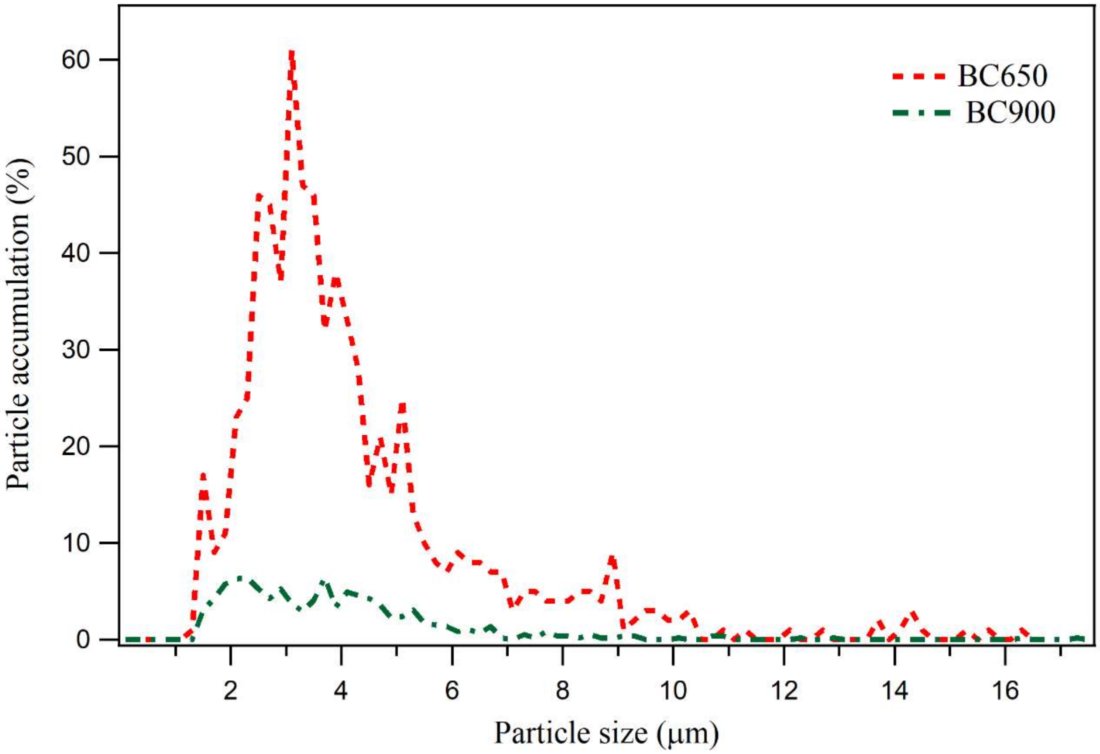

Figure S1: Particle size distribution of biocarbon ball-milled at two different pyrolysis temperature (650 and 900 ºC) obtained by image-based particle size analysis

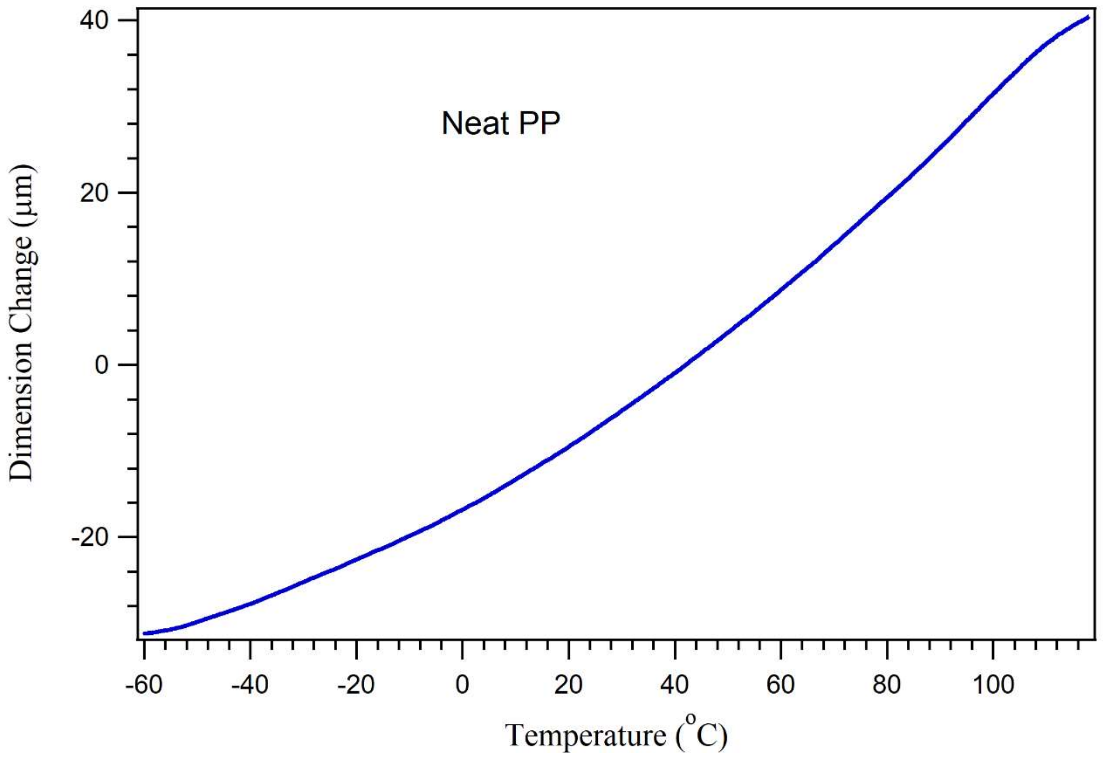

Figure S2: Typical thermal expansion behavior of neat PP in the ND during the second heating runs.
